# Thrombolysis risk prediction: applying the SITS-SICH and SEDAN scores in South African patients

**DOI:** 10.5830/CVJA-2014-043

**Published:** 2014

**Authors:** A von Klemperer, K Bateman, A Bryer, J Owen

**Affiliations:** Division of Neurology, Department of Medicine, Groote Schuur Hospital and University of Cape Town, South Africa; Division of Neurology, Department of Medicine, Groote Schuur Hospital and University of Cape Town, South Africa; Division of Neurology, Department of Medicine, Groote Schuur Hospital and University of Cape Town, South Africa; Department of Radiology, Groote Schuur Hospital and University of Cape Town, South Africa

**Keywords:** stroke, acute ischaemic stroke, thrombolysis, intracranial haemorrhage, risk, SEDAN, SITS-MOST, rTPA, recombinant tissue plasminogen activator, Safe Implementation of Treatment in Stroke – Symptomatic IntraCerebral Haemorrhage risk score, South Africa, Groote Schuur Hospital

## Abstract

At present, the only specific medical treatment for acute ischaemic stroke is intravenous administration of recombinant tissue plasminogen activator within 4.5 hours of stroke onset. In the last year, two scores for risk stratification of intracranial haemorrhage have been derived from multicentric European trial groups, the Safe Implementation of Treatment in Stroke – Symptomatic IntraCerebral Haemorrhage risk score (SITS-SICH) and the SEDAN score. The aim of this study was to pilot their use in a cohort of patients treated at a South African tertiary hospital.

Prospectively collected data were used from a cohort of 41 patients who underwent thrombolysis at Groote Schuur Hospital from 2000 to 2012. Computerised tomography brain imaging was available for review in 23 of these cases. The SITS-SICH and SEDAN scores were then applied and risk prediction was compared with outcomes.

Two patients suffered symptomatic intracranial haemorrhage (SICH), representing 4.9% (95% CI: 0–11.5%) of the cohort. This was comparable to the SICH rate in both the SITS-SICH (5.1%) and SEDAN (6.5%) cohorts. Patient scores in the Groote Schuur Hospital cohort appeared similar to those of the validation cohorts of both SITS-SICH and SEDAN.

With increasing use of thrombolysis in a resource-constrained setting, these scores represent a potentially useful tool in patient selection of those most likely to benefit from intravenous thrombolysis, reducing risk for SICH and with the added benefit of curtailing cost.

## Abstract

Stroke is the most common cause of death in people over the age of 50 years in South Africa.^1^ It is estimated that there were approximately 75 000 new cases of stroke in South Africa in 2008. Of these, approximately 25 000 were fatal within the first 28 days. In 2007, there were 350 000 people living with stroke in South Africa, of whom 35% had moderate to severe disability as a result of their stroke. 2

Currently, intravenous (IV) administration of recombinant tissue plasminogen activator (tPA) within 4.5 hours of symptom onset is the only medical therapy shown to improve outcomes in acute ischaemic stroke.^3^-^5^ It has become the standard of care in many international stroke centres.

However, it is still unclear which patients are most likely to benefit and in what treatment time frame. Initial evidence demonstrated the benefit of thrombolysis in selected patients presenting within three hours. It has subsequently been shown that the window of maximum beneficial effect extends to 4.5 hours.^6^,^7^

Careful selection of patients suitable for thrombolysis treatment is required to maximise the benefit obtained and offset the risk of clinical deterioration due to symptomatic intracranial haemorrhage. Observational data from SITS-MOST show thrombolysis to be as safe and effective in real clinical practice as in clinical trials; however, the rate of SICH remained between 1.7 and 4.6%.^8^ It has been estimated that of 100 patients treated with tPA, one will have a severely disabling or fatal outcome due to tPA-related intracranial haemorrhage. 9

In 2011, Wasserman and Bryer published data on a cohort of 42 patients treated with tPA at a tertiary hospital in Cape Town, which showed comparable safety and early outcomes to similar cohorts in both developed and developing countries.^10^ Many clinicians however remain concerned about the use of this treatment modality and the risk of SICH.^11^

Two scoring systems that attempt to stratify patients by their risk of developing SICH following thrombolysis have recently been derived from multicentre cohorts of patients – the Safe Implementation of Treatment in Stroke – Symptomatic IntraCerebral Haemorrhage risk score (SITS-SICH)^12^ and the SEDAN score.^13^ The SITS-SICH score is derived from the SITS-MOST patient cohort and was internally validated on a random sample of more than 15 000 patients; it incorporates primarily clinical variables which best predict the SICH following thrombolysis with tPA. The SEDAN score is based on both clinical and radiological findings on computerised tomography (CT) of the brain, and was externally validated in a smaller cohort of 828 patients.

Both scores however have been validated in European populations in developed countries and their utility in a different setting is not known. Accurate assessment of risk is necessary for clinicians to select patients who will most benefit from thrombolytic therapy, at the lowest risk of bleeding complications such as SICH. In a resource-constrained setting in which the cost of thrombolytic therapy is significant, the inclusion of a risk-prediction score to the protocol used for treatment may improve the cost–benefit ratio, and optimise the allocation of scarce resources.

The aim of this pilot study was to evaluate the performance of both the SITS-SICH and SEDAN scores in predicting the risk of SICH in a Groote Schuur thrombolysis stroke cohort.

## Methods

Data were extracted from a prospective cohort comprising all patients presenting to Groote Schuur Hospital (GSH) between 2000 and May 2012 with acute ischaemic stroke, who received thrombolytic therapy with IV tPA according to the GSH Stroke Unit protocol. Patients who received mechanical thrombolysis or intra-arterial tPA were excluded.

Age, gender, past medical history and prior medication were recorded. Admission data of vital signs, serum glucose levels, stroke severity according to the NIHSS, disability according to the modified Rankin score, and details of tPA administration (time to onset and dose) were also recorded. For this study, all available CT brain scans performed on admission (pre-thrombolysis) were re-evaluated by a radiologist in training for signs of early infarct or the dense middle cerebral artery (MCA) sign. The SITS-SICH and SEDAN scores (see below) were calculated from these data and patients were risk-stratified accordingly.

Both the SITS-SICH and SEDAN scoring systems (see Tables 1, 2) were developed using multiple regression analyses, and identified elevated serum glucose levels and high NIHSS scores on admission as poor prognostic indicators. The risk of SICH varied according to the SICH definition used: by the SITS-MOST definition, the risk ranged from 0.2% (score of 0) to 9.2% (score ≥ 9) while the risk of SICH by the ECASS II definition ranged from 1.4% (score 0) to 23.2% (score ≥ 9). The SEDAN score revealed an increasing risk of SICH in the external validation cohort, ranging from 0.01% (score 0) to 27.8% (highest score 6). The single largest risk factor identified for the development of SICH in the SITS-MOST study was dual antiplatelet therapy with both aspirin and clopidogrel.^12^,^13^

**Table 1:** Components of SITS score and overall risk level^12^

*Category*	*Points (15)*
Aspirin + clopidogrel therapy	2
Aspirin monotherapy	1
NIHSS > 13	2
NIHSS 7–12	1
Blood glucose ≥ 180 mg/dl*	2
Age ≥ 72 years	1
Systolic BP ≥ 146 mmHg	1
Weight ≥ 95 kg Onset-to-treatment time ≥ 180 min	1
History of hypertension	1

*180 mg/dl ≈10 mmol/l

**Table 2:** SEDAN score^13^

*Category*	*Total*	*6*
Blood sugar (glucose) on admission	≤ 8 mmol/l	0
	8.1–12 mmol/l	1
	> 12 mmol/l	2
Signs of early infarction on admission CT*	No	0
	Yes	1
Dense middle cerebral artery sign on admission CT	No	0
	Yes	1
Age (years)	≤ 75	0
	> 75	1
NHSS score on admission	0–9 points	0
	≥ 10 points	1

*Signs of early infarction: hypo-attenuation of the middle cerebral artery territory (< 1/3), obscuration of the lentiform nucleus, cortical sulcal effacement, focal hypo-attenuation, loss of the insular ribbon/obscuration of the Sylvian fissure, loss of grey–white differentiation in the basal ganglia, hypo-attenuation of the basal ganglia.

The primary outcome was symptomatic intracranial haemorrhage according to either the SITS-MOST and/or ECASS II definitions. The SITS-MOST definition of SICH is a local or remote type II parenchymal haemorrhage within 22 to 36 hours after treatment (or sooner) associated with a ≥ fourpoint deterioration on the NIHSS score from baseline or from the lowest score from baseline to 24 hours, or leading to death.^12^

The ECASS II definition of SICH was any intracranial haemorrhage on any post-treatment image, within seven days of initiating treatment associated with a ≥ four-point deterioration on the NIHSS score from baseline or from the lowest score in seven days, or leading to death.^14^ Other outcomes reported were death, asymptomatic intracranial haemorrhage (AIH), and extracranial haemorrhage (EH).

During most of the cohort period, the GSH Stroke Unit protocol required post-thrombolysis CT brain scans to be performed routinely within 48 hours of thrombolysis, or urgently with any suspicion of an intracerebral haemorrhage. Post-thrombolysis CT scans were reviewed for this study by a radiologist trainee blinded to clinical outcomes for evidence of intracranial haemorrhage.

Ethical approval was obtained from the UCT Groote Schuur Hospital human research ethics committee (Ref: 499/2013).

## Results

In total, 45 patients underwent thrombolysis for acute ischaemic stroke at the GSH Stroke Unit from January 2000 to May 2012. Four patients underwent mechanical thrombolysis with intraarterial tPA, and were excluded. The remaining 41 patients who received IV tPA for acute ischaemic stroke were included (see Table 3).

**Table 3:** Baseline characteristics

*Baseline characteristics (n = 42)*	
Median age, years (IQ range)	62 (50–66)
Weight on admission, kg (IQ range)	76 (67–80)
Preceding history of hypertension, *n* (%)	27 (66)
Median systolic BP on admission, mmHg (IQ range)	149 (134–175)
On anti-platelet therapy at admission, *n* (%)	11 (27)
Abnormal serum glucose on admission, *n* (%)	8 (20)
Mean time to thrombolysis (min)	169
Median NIHSS score on admission, *n* (IQ range)	14 (11–17)
CT brain scans (*n* = 23)	
Early signs of infarction, *n* (%)	16 (70)
Hyperdense MCA, *n* (%)	7 (30)

Five patients were older than 72 years, and two patients were older than 75 years at stroke onset. Admission systolic blood pressures (SBP) were above 180 mmHg in five patients and greater than 220 mmHg in one patient. Of those taking antiplatelet therapy at the time of admission, all were on aspirin monotherapy. Time to onset of treatment with tPA was greater than 180 min in 13 patients and none of these were treated more than 4.5 hours after onset of symptoms. Pre- and post-thrombolysis CT scans were available for review in 23 patients.

Two patients suffered SICH (ECASS II definition) postthrombolysis, comprising 4.9% of the cohort. CT scans were available for review in one patient only and confirmed that the haemorrhage fulfilled the SITS-MOST criteria (2.4%). Of the four patients who died during their admission for stroke, one patient suffered a fatal SICH, while three other patients died from causes unrelated to thrombolysis therapy: cardiogenic heart failure following an acute myocardial infarction, and recurrent cerebral infarction and subsequent pneumonia. Eight patients had evidence of asymptomatic intracranial haemorrhage, attributed to haemorrhagic conversion of the ischaemic infarct. In four of these patients, the CT scan was available to verify this finding. Two patients had extracranial haemorrhage, including one patient with a hip haematoma.

The median SITS-SICH score for our patient cohort was 4 (IQR 2–5). The patients were stratified into low, average, moderate and high risk (see Table 1). In the GSH cohort, the majority of patients had 3–5 points, or average risk, including the two patients who developed SICH. There were no patients who were scored as high risk (> 9). The distribution of patient risk in the GSH cohort differed from the SITS-SICH cohort, with more patients classified as low or average risk, and no high-risk patients (Table 4).

**Table 4:** Comparison of SEDA N scores and risk of SICH by ECASS II definition for GSH and SEDAN validation cohorts

*Score*	*Total GSH cohort (n = 41) % (n)*	*Total SITS cohort (n = 15 814) % (n)*	*SICH rate (GSH) %*	*SICH rate (SITS) %*
Low (0–2 points)	29 (12 /41)	22.7	0	1.6
Average (3–5 points)	53.7 (22/41)	55	9	4.7
Moderate (6–8 points)	17.1 (7/41)	21.4	0	8.9
High (> 9 points)	0	1.1	0	23.2
Overall rate			4.6 (2/41)	5.1

One GSH patient who had a SEDAN score of 3 suffered a SICH (by both ECASS II and SITS-SICH criteria). There was one death in this group, due to complications related to pneumonia (Table 5).

**Table 5:** Comparison of SEDA N scores and risk of SICH by ECASS II definition for GSH and SEDAN validation cohorts

*Score*	*Total GSH cohort (n = 23) % (n)*	*Total SEDAN cohort %*	*SICH rate (GSH) (n = 2) % (n)*	SICH rate (SEDAN)* %
0	4.4 (1)	12.4	0	0.9
1	30.4 (7)	27.5	0	3.5
2	34.8 (8)	28.3	0	5.1
3	26.1 (6)	20.9	16.7 (1)	9.2
4	0 (0)	8.6	0	16
5	4.4 (1)	2.2	0	27
6	0	0	0	0

## Discussion

Urgent thrombolysis with IV tPA is a priority in the emergency medical treatment of acute ischaemic stroke. Robust efficacy data exists for this therapeutic modality, particularly when administered within the first 90 min, and the window of benefit has widened since it was first introduced from three to 4.5 hours after onset of symptoms. Published data from the GSH Stroke Unit reveal similar rates of SICH to those from developed and several developing countries, which provide some reassurance to clinicians concerned about the safety profile of this modality in a South African setting. However, the risk of SICH remains, and care should be taken to select patients who are likely to have the most benefit at the lowest risk of SICH.

To be practical, the application of risk scores for SICH should include information that is easily obtained in the emergency unit (EU). They should contain independent risk factors for SICH and take into account the interplay between these factors in an individual patient.

The SITS-SICH score uses clinical variables that can be attained relatively quickly and easily at the bedside in a resource-constrained area. It has been validated in over 16 000 patients from multiple centres, many of whom did not have prior experience in thrombolysis. The SEDAN score uses clinical information but it relies on the assessment of brain CT imaging for subtle signs of stroke, which may be overlooked in a busy EU setting by inexperienced reviewers. Many South African centres use older (fewer slice) scanners that would decrease the sensitivity in detecting such signs.

The overall rates of SICH seen in the SITS-SICH validation cohort of 5.1% per ECASS II definition and 1.8% per SITSMOST definition compare with the GSH rates of 4.8 and 2.4%, respectively. The SICH rate in the SEDAN score validation cohort was 6.5%. There appeared to be a trend towards GSH patients being slightly lower risk than either of the SITS-MOST or SEDAN validation cohorts. This may reflect the more cautious approach in patient selection being used at our centre.

The main limitation of this pilot study was that of small sample size and low event rate in the GSH cohort. One is unable to comment on the ability of either score to reliably predict the risk of haemorrhage. However, the overall rate of SICH by the ECASS II definitions was similar between the cohorts studied.

A further limitation was that CT brain scans taken prior to 2003 were not available for a review of the images, although reports were present. Therefore, signs of early infarction and a dense middle cerebral artery sign could not be evaluated as is required for the SEDAN scoring system, nor could we confirm the presence of a type II parenchymal haemorrhage, required for the SITS-MOST definition of SICH.

## Conclusion

The scores, in particular the SITS-SICH score, represent a potentially useful clinical tool to aid in patient selection for thrombolysis in ischaemic stroke. This study, piloting their use in a South African cohort, suggests that they may be applicable in our context but further research is required to validate their use. With the increasing use of thrombolysis on a national level, such risk-stratification tools might be considered for inclusion into a stroke unit protocol.
